# Fusional Vergence Detected by Prism Bar and Synoptophore in Chinese Childhood Intermittent Exotropia

**DOI:** 10.1155/2015/987048

**Published:** 2015-04-14

**Authors:** Tao Fu, Jing Wang, Moran Levin, Qing Su, Dongguo Li, Junfa Li

**Affiliations:** ^1^Beijing Tongren Eye Centre, Beijing Tongren Hospital, Capital Medical University, Beijing Ophthalmology and Visual Science Key Lab, Beijing 100069, China; ^2^Department of Ophthalmology, University of Maryland Medical Center, Baltimore, MD 21201-1595, USA; ^3^Department of Neurobiology and Beijing Institute for Brain Disorders, Capital Medical University, Beijing 100069, China

## Abstract

*Purpose*. To measure the changes in fusional vergence in Chinese children with intermittent exotropia (IXT) and the association with the control of IXT. *Methods*. Ninety-two patients with IXT (8–15 years old) were compared with 86 controls. Exodeviation control was evaluated using the Revised Newcastle Control Score. Angle of deviation was measured using prism and alternate cover testing at distance and near. Fusional vergence was measured using prism bar and synoptophore. This study was registered with ChiCTR-RCC-13003920. *Results*. Using prism bar, convergence break points were lower whereas divergence break points were higher in children with IXT at distance (*P* < 0.001) and near (*P* < 0.001) compared with controls. There was no significant difference in mean divergence amplitudes between the two groups when testing using a synoptophore (*P* = 0.53). In children with IXT, the distance between recovery point and break point in both convergence (distance: *P* = 0.02; near: *P* = 0.02) and divergence (distance: *P* < 0.001; near: *P* < 0.001) was larger than controls when detected by prism bar and synoptophore (convergence: *P* = 0.005; divergence: *P* = 0.006). *Conclusions*. Children with IXT have reduced convergence amplitudes as detected by both prism bar and synoptophore.

## 1. Introduction

Intermittent exotropia (IXT) accounts for 44.9–72% of patients with primary horizontal strabismus in Asia [1–3]. It typically begins as an exophoria that progresses to an intermittent deviation and that may then deteriorate into a constant exodeviation in up to 75% of cases [4]. In IXT, control of the exodeviation may rely on fusional vergence.

Motor fusional vergence is the ability to maintain binocular vision through a range of induced vergences (often prism-induced) until a point is reached at which the binocular vision is interrupted and the patient experiences diplopia. The normal range of fusional vergence has been described since 1948 [5–8], but only in recent years fusional vergence in IXT has been studied and reported. In recent years, several studies have been published comparing the differences between convergence and divergence of patients with IXT and normal subjects using various methods, and these studies have shown conflicting results [9–11]. Sharma et al. found that both convergence and divergence amplitudes were decreased in patients with IXT [11]. In their study, fusional vergence was measured using prisms to neutralize the strabismic deviation. Two other studies determined vergence from a state of spontaneous binocular fusion and have reported subnormal convergence reserves at distance [9] and normal near fusional divergence but reduced distance fusional divergence [10].

In Western populations, it has been found that esotropia is twice as common as exotropia; however, recent studies have shown that exotropia is far more common in Asian populations [1–3]. Therefore, the present study was designed to measure the changes in fusional vergence in patients with IXT using a prism bar and synoptophore in Chinese children, to compare the values detected by the two methods, and to evaluate the correlations between the vergence and the control of IXT.

## 2. Materials and Methods

This was a prospective observational study. The research protocol and informed consent form were approved by the Institutional Review Board of the Beijing Tongren Hospital. Written informed consent was obtained from a parent or legal guardian for each participant. The study was registered with the Chinese Clinical Trial Registry (http://www.chictr.org/en/; ChiCTR-RCC-13003920).

### 2.1. Participants

Ninety-two consecutive patients with IXT at distance and near, aged between 8 and 15, were evaluated at the outpatient clinic of the Beijing Tongren Hospital between July 2013 and July 2014 and were included in the study. Eighty-six asymptomatic age- and gender-matched normal subjects were also recruited during the same period as controls.

Inclusion criteria were (1) IXT with exodeviation of at least 10 prism diopters (pd) at distance and near and (2) best corrected visual acuity of 6/6 or better in each eye. Exclusion criteria were (1) amblyopia; (2) >2 diopters of anisometropia; (3) incomitance of the horizontal or vertical deviation; (4) vertical deviation of ≥5 pd; or (5) significant oblique muscle overaction. Cases with convergence insufficiency-type IXT (near angle >10 pd greater than distance) or those with a previous history of strabismus surgery were also excluded. A patient was excluded if he was manifest at distance, in which condition the vergence could not be determined by using prism bar.

### 2.2. Testing Procedure

*Angle of Deviation*. All subjects underwent a complete ophthalmic and orthoptic assessment including prism and alternate cover testing, prism bar worth four-dot test, striated glasses test of Bagolini, and TNO test. Clinical testing was performed using best refractive correction. The angle of deviation was measured using prism and alternate cover testing at distance (6 m) and near (33 cm) in the primary position with fixation on an accommodative target.

*Control of Exodeviation*. The control of the exodeviation was quantified using the Revised Newcastle Control Score (RNCS) (Buck et al., 2008) [12] (Table 1). Briefly, the scoring of the home control item ranges from 0 to 3. The clinical control score, which is assessed for both near and distance, ranges between 0 and 3. The RNCS results in a 10-point scale ranging from 0 to 9, with higher scores indicating increasing severity.

*Fusional Vergence*. The fusional vergence was assessed using a 1 to 40 pd horizontal prism bar for both distance (6 m) and near fixations (33 cm). A single Snellen letter (6/12 level) was used for both distance and near fixations. Prism strength was increased gradually and patients were asked to report when the fixation object appeared to double. The prism power was noted as the break point and this was confirmed by the examiner as an exotropia developing with no subsequent recovery of motor fusion. The prism power was then gradually decreased, and the point at which the patient regained single vision was recorded as the recovery point. Both convergence and divergence break points and recovery points were measured with base-out (BO) prism and base-in (BI) prism, respectively. For subjects who retained fusion at 40 pd (3 of 28 patients at near, 6 of 26 normal subjects at near), the break points were assigned 45 pd for analysis purposes and they were excluded from the recovery analysis. The blur point was not consistently recorded due to the difficulty in obtaining such data when assessing young children. The testing order for fusional vergence was distance BI, near BI, distance BO, and near BO.

Fusional convergence and divergence amplitudes were detected using a synoptophore L-2510B/L-2510HB (Inami & Co., Ltd., Japan) approximately 1 hour after the prism bar examination to allow sufficient time for recovery of fusion. Horizontal fusional vergence was measured with fusion slides subtending a visual angle of 6 degrees horizontally and 8 degrees vertically. The break points and recovery points were measured using the synoptophore and were recorded in prism diopters.

For the present study, we used the following definitions in order to simplify the description of the parameters detected by prism bar and synoptophore. The values of break points measured by prism bar were defined as the amplitude of the vergence, which was defined as convergence reserve in the study by Hatt et al. [9]. The amplitude of vergence on synoptophore was defined as the distance between the break points and the points of simultaneous perception. The amplitudes of convergence and divergence on synoptophore were calculated as the break points minus the points of simultaneous perception. The ease of recovery was defined as the distance between the break point and recovery point and was calculated by subtracting the break point from the recovery point.

### 2.3. Statistical Analysis

Statistical analysis was performed using SPSS 13.0 for Windows (SPSS Inc., Chicago, IL, USA). Mean vergence amplitudes and the distance between fusional recovery and break points were calculated for children with IXT and normal subjects. Independent sample Student's *t*-test was used for comparison of various parameters between cases and normal controls. Correlations of control score with vergence amplitudes detected by prism bar and synoptophore were calculated using the Spearman rank correlation. *P* values ≤0.05 were regarded as statistically significant.

## 3. Results

Ninety-two consecutive cases of IXT were included in our study. Eighty-six age- and gender-matched controls were evaluated. The mean age of the patients was 10.29 ± 1.53 (range 8 to 14) years and that of controls was 10.55 ± 2.30 (range 8 to 15) years (*P* = 0.67). There were 49 males (53.26%) and 43 females (46.74%) in the IXT group and 48 males (55.81%) and 38 females (44.19%) in the control group (*P* = 0.79). There was no significant difference in refraction between the two groups (*P* = 0.61 in the right eye and *P* = 0.38 in the left eye) (Table 2). The mean amount of deviation (pd) in patients with IXT was 38.25 ± 14.83 (range 10 to 70) at near and 36.67 ± 15.69 (range 15 to 70) at distance (*P* = 0.57).

### 3.1. Fusional Vergence Detected by Prism Bar

**Figure 1:**
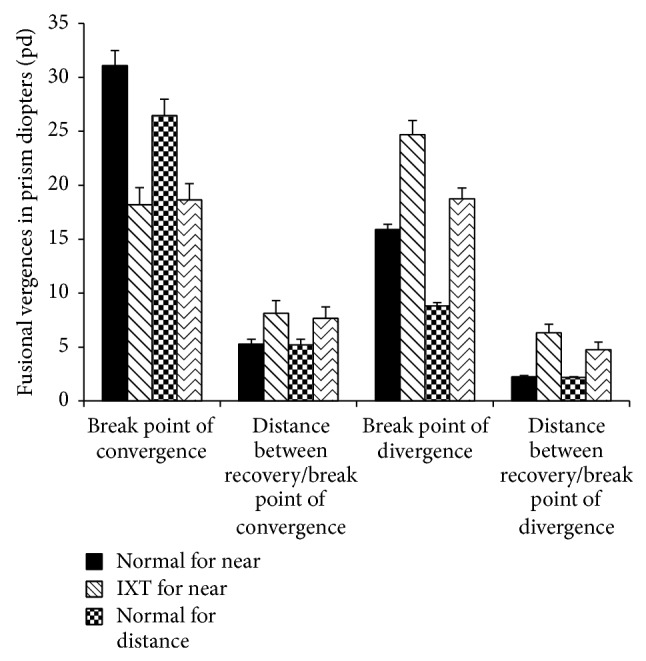
Comparison of the fusional vergence detected using a prism bar in patients with IXT and normal subjects. The mean convergence amplitude was significantly lower for children with IXT compared with normal children at both distance and near (distance: 18.65 ± 1.50 versus 26.46 ± 1.53 pd, *P* < 0.001; near: 18.20 ± 1.59 versus 31.08 ± 1.40 pd, *P* < 0.001). The mean divergence amplitudes were significantly greater for children with IXT than for normal children (distance: 18.75 ± 0.99 versus 8.81 ± 0.32 pd, *P* < 0.001; near: 24.69 ± 1.33 versus 15.91 ± 0.46 pd, *P* < 0.001). The mean distance between the recovery and break points for both convergence and divergence was significantly larger at both distance and near for IXT children compared with normal subjects (for convergence, distance: 7.67 ± 1.06 versus 5.21 ± 0.51 pd, *P* = 0.02; near: 8.13 ± 1.19 versus 5.30 ± 0.44 pd, *P* = 0.02; for divergence, distance: 4.76 ± 0.72 versus 2.18 ± 0.08 pd, *P* < 0.001; near: 6.33 ± 0.79 versus 2.26 ± 0.10 pd, *P* < 0.001).

We detected fusional convergence and divergence amplitudes using a prism bar and synoptophore. Using the prism bar, the mean convergence amplitudes were significantly lower for children with IXT compared with normal children at both distance and near (distance: 18.65 ± 1.50 versus 26.46 ± 1.53 pd, *P* < 0.001; near: 18.20 ± 1.59 versus 31.08 ± 1.40 pd, *P* < 0.001, Figure 1). The mean distance between recovery and break points for convergence was significantly larger at both distance and near for children with IXT compared with normal subjects (distance: 7.67 ± 1.06 versus 5.21 ± 0.51 pd, *P* = 0.02; near: 8.13 ± 1.19 versus 5.30 ± 0.44 pd, *P* = 0.02, Figure 1). The mean divergence amplitudes were significantly greater for children with IXT than those for normal children (distance: 18.75 ± 0.99 versus 8.81 ± 0.32 pd, *P* < 0.001; near: 24.69 ± 1.33 versus 15.91 ± 0.46 pd, *P* < 0.001, Figure 1). The mean distance between recovery and break points for divergence at both distance and near was also significantly greater for children with IXT than for normal children (distance: 4.76 ± 0.72 versus 2.18 ± 0.08 pd, *P* < 0.001; near: 6.33 ± 0.79 versus 2.26 ± 0.10 pd, *P* < 0.001, Figure 1).

### 3.2. Fusional Vergence Detected by Synoptophore

**Figure 2:**
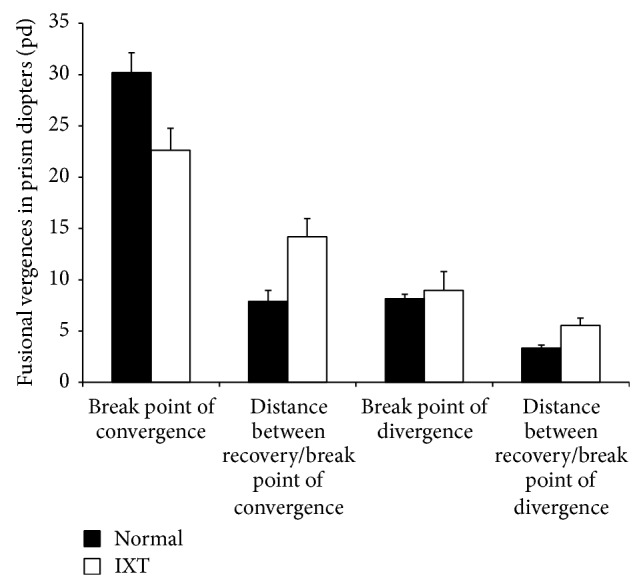
Comparison of fusional vergence detected using a synoptophore between patients with IXT and normal subjects. The mean convergence amplitudes were significantly lower for children with IXT compared with normal children (22.62 ± 2.15 versus 30.19 ± 1.95 pd, *P* = 0.01). No significant difference was observed in the divergence amplitude between children with IXT and normal children (8.98 ± 1.82 versus 8.15 ± 0.44 pd, *P* = 0.53). The distance between recovery and break points for both convergence and divergence was larger for patients with IXT compared with normal controls (convergence: 14.18 ± 1.79 versus 7.89 ± 1.09 pd, *P* = 0.005; divergence: 5.56 ± 0.71 versus 3.36 ± 0.28 pd, *P* = 0.006).

Using synoptophore, the convergence amplitudes were significantly lower for children with IXT compared with normal children (22.62 ± 2.15 versus 30.19 ± 1.95 pd, *P* = 0.01, Figure 2). Divergence amplitudes were slightly greater for children with IXT than for normal children, although no significant difference was found (8.98 ± 1.82 versus 8.15 ± 0.44 pd, *P* = 0.53, Figure 2). The distance between recovery and break points for both convergence and divergence was larger for patients with IXT than for normal controls (convergence: 14.18 ± 1.79 versus 7.89 ± 1.09 pd, *P* = 0.005; divergence: 5.56 ± 0.71 versus 3.36 ± 0.28 pd, *P* = 0.006, Figure 2).

### 3.3. Correlation of Vergence and Exotropia

When measured using the prism bar, there was a moderate inverse correlation between convergence amplitudes and control score at distance (*r* = −0.57, *P* = 0.002) and at near (*r* = −0.63, *P* < 0.001, Table 3). We did not observe any significant correlation between control score and divergence amplitudes for near (*r* = 0.08, *P* = 0.71) or distance (*r* = 0.28, *P* = 0.12, Table 3).

## 4. Discussion

In the present study, we measured fusional vergence both by using the standard clinical method of progressively increasing prismatic power from a state of spontaneous binocular fusion and by using a synoptophore to neutralize the strabismic deviation. At both distance and near, the mean convergence amplitudes were significantly lower for children with IXT than those for normal children using both methods. When measured with a prism bar, the mean divergence amplitudes were significantly greater for children with IXT than for normal children. There was no significant difference in mean divergence amplitudes between the two groups when tested using a synoptophore. A moderate inverse correlation was found between convergence amplitudes and control score at both distance and near. However, no significant correlation was found between the control score and the divergence amplitudes for distance or near.

Hatt et al. have recently reported that the mean convergence break points (defined as convergence reserve) were significantly lower at distance fixation for children with IXT compared with orthophoric children [9]. Nevertheless, they found that near convergence break points were comparable to visually normal children, but we observed reduced convergence break points at both distance and near, in accordance with the study by Sharma et al. [11]. The mean near convergence break point values in subjects with IXT were similar in the present study compared with these two studies. The normal reference values were similar in the present study (31 pd) and the Sharma study (28 pd), but both were higher than those used in Hatt's study (18 pd) (Hatt et al., 2011) [9], potentially explaining the differences in results.

Various studies have found variability in reporting divergence break points. Sharma et al. reported reduced divergence amplitudes in IXT subjects that differed from the results of our present study [11]. Liebermann et al. have reported that most children with IXT have normal near fusional divergence, but that nearly half had reduced distance fusional divergence [10]. They found that the distribution of fusional divergence break points in IXT was normal at near but bimodal at distance. However, our data showed that the distribution of values of divergence break points was normal. Rowe found that exophoric subjects had a trend towards larger divergence amplitudes, which to some extent supports the results of the present study [13].

In the present study, in addition to the traditional prism bar method, we detected fusional vergence using a synoptophore. In contrast to the prism bar method in which the subject begins in a state of spontaneous binocular fusion, the strabismic deviation is first neutralized when assessing fusional vergence by synoptophore. When detected by synoptophore, the mean convergence amplitudes were significantly lower for children with IXT compared with normal children, which is similar to the results observed by the prism bar. However, we did not observe a significant difference in mean divergence amplitudes between patients with IXT and normal subjects, which is different from the findings detected by prism bar. The difference of the smooth (synoptophore) and step (prism bar) vergence testing might be a reason for the different results between the two methods [14, 15]. The difference in binocular state during the assessment, a state of spontaneous binocular fusion (prism bar) versus a state of neutralization of the deviation of the intermittent exotropia (synoptophore), might be another important factor. Jampolsky has postulated that, in IXT, binocular alignment is achieved by convergence mechanisms and that, therefore, when assessed from a point of spontaneous fusion, BI prisms initially result in a relaxation of convergence or “deconvergence” until the deviation is neutralized [16]. On the other hand, using the synoptophore, the deviation is neutralized first; the divergence detected does not contain the components of “deconvergence” and might reflect the true fusional divergence amplitudes in patients with IXT. In addition, the patients were already using motor fusion to control their deviation, which can lead to an apparent abnormal BO range in larger deviations. The similarity in divergence amplitudes between patients with IXT and normal subjects indicates that convergence is more involved in the control of IXT in that the fragility of fusion is due to reduced convergence amplitudes rather than the enlarged divergence amplitudes.

The distance measured between the recovery and break points using both the prism bar and synoptophore was significantly larger in IXT patients compared with normal controls. Our data indicated that the patients with intermittent deviations often have relatively poor ability to recover fusion.

There was a strong correlation between the convergence break points detected using the prism bar and the control score at distance and near, similar to the study by Hatt et al. [9]. Another study in Chinese children showed similar results [17]. There was no significant correlation between divergence amplitude detected by prism bar and control score, which is similar to the findings by Liebermann et al. (*r* = −0.29, *P* = 0.11) [10].

In children with IXT, some factors may influence the fusional convergence ability such as fixation distance, the type of visual stimulus, the size of the target, the type of visual environment, and long-term adaptation [13, 18–20]. It should be noted that testing conditions were uniform between all subjects in the present study. Therefore, differences between subjects should not be attributed to differences in the testing environment.

## 5. Conclusions

In conclusion, we observed that in Chinese children with IXT, the mean convergence break points were lower and that the fusional recovery was poorer compared with normal controls, as detected using both prism bar and synoptophore. Of interest, the mean divergence break points of patients with IXT were comparable with those of normal subjects when detected with synoptophore but significantly larger when measured with the prism bar. This may support a “deconvergence” mechanism of IXT and indicates that convergence is involved in the control of IXT.

## Figures and Tables

**Table 1:** The revised Newcastle Control Score.

	Score
Homing control	
XT or monocular eye closure seen	
Never	0
<50% of time fixing at distance	1
>50% of time fixing at distance	2
>50% of time fixing at distance + seen at near	3
Clinical control	
Near	
Immediate realignment after dissociation	0
Realignment with aid of blink or refixation	1
Remains manifest after dissociation or prolonged fixation	2
Manifest spontaneously	3
Distance	
Immediate realignment after dissociation	0
Realignment with aid of blink or refixation	1
Remains manifest after dissociation or prolonged fixation	2
Manifest spontaneously	3
Total: NCS = home + near + distance	

XT: exotropia.

**Table 2:** Characteristics of patients with intermittent XT and normal subjects.

	IXT	Normal control	*t*-value	*P* value
Number of cases	92	86		
Mean age (years)	10.29 ± 1.53	10.55 ± 2.30	−0.45	0.67
Gender				
Male	49	48	0.09 (*x* ^2^ value)	0.79
Female	43	38		
Refraction				
Right eye	−1.95 ± 1.63	−1.68 ± 1.79	−0.52	0.61
Left eye	−2.01 ± 1.73	−1.57 ± 1.51	−0.89	0.38

**Table 3:** Correlation between control of deviation and fusional vergence detected using a prism bar.

	Control score	
	*R*-value	*P* value
Convergence breakpoints for near	−0.63	<0.001
Convergence breakpoints for distance	−0.57	0.002
Divergence breakpoints for near	0.08	0.71
Divergence breakpoints for distance	0.28	0.12
Distance between convergence recovery/break points for near	−0.05	0.76
Distance between convergence recovery/break points for distance	−0.07	0.71
Distance between divergence recovery/break points for near	0.08	0.62
Distance between divergence recovery/break points for distance	0.22	0.21
